# Influence of Transfusion on the Risk of Acute Kidney Injury: ABO-Compatible versus ABO-Incompatible Liver Transplantation

**DOI:** 10.3390/jcm8111785

**Published:** 2019-10-25

**Authors:** Je Hyuk Yu, Yongsuk Kwon, Jay Kim, Seong-Mi Yang, Won Ho Kim, Chul-Woo Jung, Kyung-Suk Suh, Kook Hyun Lee

**Affiliations:** 1 Department of Anesthesiology and Pain Medicine, Seoul National University Hospital, Seoul National University College of Medicine, Seoul 03080, Korea; jehyukyu@gmail.com (J.H.Y.); ulsan-no8@hanmail.net (Y.K.); jaykim.medicine@gmail.com (J.K.); seongmi.yang@gmail.com (S.-M.Y.); jungcwoo@gmail.com (C.-W.J.); leekh@snu.ac.kr (K.H.L.); 2 Department of Surgery, Seoul National University College of Medicine, Seoul 03080, Korea; kssuh@snu.ac.kr

**Keywords:** acute kidney injury, liver transplantation, living donor, ABO blood group system, blood transfusion

## Abstract

ABO-incompatible liver transplantation (ABO-i LT) is associated with a higher risk of acute kidney injury (AKI) compared to ABO-compatible liver transplantation (ABO-c LT). We compared the risk of AKI associated with transfusion between ABO-c and ABO-i living donor liver transplantation (LDLT). In 885 cases of LDLT, we used a propensity score analysis to match patients who underwent ABO-c (*n* = 766) and ABO-i (*n* = 119) LDLT. Baseline medical status, laboratory findings, and surgical- and anesthesia-related parameters were used as contributors for propensity score matching. AKI was defined according to the “Kidney Disease Improving Global Outcomes” criteria. After 1:2 propensity score matching, a conditional logistic regression analysis was performed to evaluate the relationship between the intraoperative transfusion of packed red blood cells (pRBCs) and fresh frozen plasma (FFP) on the risk of AKI. The incidence of AKI was higher in ABO-i LT than in ABO-c LT before and after matching (after matching, 65.8% in ABO-i vs 39.7% in ABO-c, *p* < 0.001). The incidence of AKI increased in direct proportion to the amount of transfusion, and this increase was more pronounced in ABO-i LT. The risk of pRBC transfusion for AKI was greater in ABO-i LT (multivariable adjusted odds ratio (OR) 1.32 per unit) than in ABO-c LT (OR 1.11 per unit). The risk of FFP transfusion was even greater in ABO-i LT (OR 1.44 per unit) than in ABO-c LT (OR 1.07 per unit). In conclusion, the association between transfusion and risk of AKI was stronger in patients with ABO-i LT than with ABO-c LT. Interventions to reduce perioperative transfusions may attenuate the risk of AKI in patients with ABO-i LT.

## 1. Introduction 

The incidence of acute kidney injury (AKI) is as high as 68% after liver transplantation (LT) for both living and deceased donor liver transplantation [1,2,3,4,5,6,7,8,9,10,11]. AKI is regarded as clinically relevant morbidity after LT due to its association with poor graft survival, increased mortality [3,4,7,12,13], and the development of chronic kidney disease [14,15].

ABO-incompatible liver transplantation (ABO-i LT) is considered to be a good therapeutic option due to limited donor selection for end-stage liver disease [16]. Currently, ABO-i LT comprises as high as 20% of total incidences of living donor LT in Asian hospitals [17], and graft survival after ABO-i LT is comparable to ABO-compatible liver transplantation (ABO-c LT) [16,18,19,20]. However, the incidence of AKI has been reported to be higher in ABO-i LT than in ABO-c LT [9]. Therefore, it is important to find the potentially modifiable predictors of AKI in ABO-i LT to mitigate the risk of post-transplant AKI. 

In ABO-i LT, the influence of intraoperative transfusion on the risk of AKI may be different from that in ABO-c LT due to prevalent metabolic alkalosis and thrombocytopenia [21]. Furthermore, recipients already receive units of fresh frozen plasma (FFP) before transplantation surgery. Therefore, the association between FFP transfusion and AKI may be stronger in patients with ABO-i LT [22].

To test this hypothesis, in this retrospective observational study, we investigated whether the incidence of AKI was higher in ABO-i LT compared to ABO-c LT and whether the influence of transfusions on post-transplant AKI differed between patients undergoing ABO-c and ABO-i LT. We hypothesized that the risk of AKI was higher in ABO-i LT and that the dose–response association between transfusion and AKI was stronger in ABO-i LT than in ABO-c LT. We sought to reduce the effects of potential confounding factors through propensity-score-matched analysis between patients undergoing ABO-c and ABO-i LT. 

## 2. Materials and Methods

### 2.1. Study Design

This retrospective observational study was approved by the institutional review board (IRB) of Seoul National University Hospital (1905-091-1034). We retrospectively reviewed the electronic medical records of 948 consecutive patients who underwent elective living donor liver transplantation (LDLT) at our institution between January 2010 and December 2018. The need for written informed consent was waived by the IRB given the study’s retrospective design. Patients with preoperative renal dysfunction, including preoperative hepatorenal syndrome type 1 (*n* = 32) and chronic kidney disease stage 3a (<60 mL/min/1.73 m^2^) or higher (*n* = 31), were excluded. The remaining 885 cases were analyzed.

### 2.2. Anesthesia, Surgical Technique, and Preoperative Desensitization Preparation

Anesthesia was maintained with propofol with remifentanil. The piggyback technique was used to anastomose the graft and donor vessels. End-to-end anastomosis of the hepatic artery and duct-to-duct anastomosis of the bile duct were performed in succession. All patients undergoing ABO-i LDLT received a single intravenous dose of rituximab 2–3 weeks prior to surgery. A plasma exchange was performed to achieve an isoagglutinin titer of 1:8 or less before the surgery and was continued until this desired titer was achieved. During the anhepatic period, intravenous methylprednisolone was administered. Immunosuppression after ABO-i LT was comprised of corticosteroid, tacrolimus, and mycophenolate mofetil [19,23]. The immunosuppressive regimen for patients undergoing ABO-c LT consisted of basiliximab induction, corticosteroid, and tacrolimus. Further details are reported in the Supplementary Materials, Section 1.

### 2.3. Data Collection

Demographic or perioperative parameters associated with postoperative renal dysfunction were collected [2,3,6,7,24,25,26,27]. Preoperatively, a Model for End-Stage Liver Disease (MELD) score and a Child–Turcotte–Pugh classification were determined for all patients [28]. History of hypertension, diabetes mellitus, ABO blood type incompatibility, warm ischemic time, cold ischemic time, graft-to-recipient body weight ratio (GRWR), operation time, intraoperative transfusion amount, postreperfusion syndrome, and crystalloid and colloid administration were collected. 

The primary outcome variable was postoperative AKI, defined according to the “Kidney Disease Improving Global Outcomes (KDIGO)” criteria [29], which have been investigated in liver transplantation [1,15]. We defined postoperative AKI based on the postoperative increase of serum creatinine (stage 1: ≥0.3 mg/dL increase within 48 h or 1.5–1.9; stage 2: 2–2.9; stage 3: more than a 3-fold increase from the baseline within the first 7 days after transplantation. The most recent serum creatinine measured before surgery was used as a baseline. Other postoperative clinical outcome variables included postoperative hemodialysis, length of intensive care unit (ICU) stay, and length of hospital stay. 

### 2.4. Statistical Analysis

SPSS software version 25.0 (IBM Corp., Armonk, NY, USA) and STATA/MP version 15.1 (StataCorp, College Station, TX, USA) were used to analyze the data. For all analyses, *p* < 0.05 was considered statistically significant. The Shapiro–Wilk test was used to determine the normality of the data. Fisher’s exact test or the chi-squared test were used to compare the incidence variables shown in Table 1. Comparisons of the continuous variables shown in Table 1 were performed using the Mann–Whitney *U* test. Missing data were present in <5% of records. Missing values of continuous variables were replaced by sex- and age-specific median values, and incidence data were assigned the most frequent sex- and age-specific values.

A multivariable logistic regression analysis was performed in all patients before matching to evaluate whether the ABO-i LT and transfusion amounts of packed red blood cells (pRBCs) and FFP were independent predictors of AKI. All potential predictors were considered in the logistic regression analysis, and a backward Wald variable selection process was used with a significance criterion of 0.20. 

To compare the incidence of AKI and the risk of transfusion between patients with ABO-c and ABO-i LT after adjusting for potential confounders, a 1:2 (ABO-i/ABO-c) propensity-score-matching analysis was performed. The following variables were used for propensity score matching: age, sex, body mass index, history of hypertension, diabetes mellitus, MELD score, alcoholic liver cirrhosis, hepatitis B, hepatitis C, hepatocellular carcinoma, preoperative hemoglobin concentration, preoperative serum creatinine, preoperative sodium concentration, preoperative left ventricular ejection fraction, the year of surgery, operation time, GRWR, graft ischemic time, and occurrence of postreperfusion syndrome. These contributors were selected because these variables have been reported to be associated with postoperative renal function [2,3,6,7,24,25,26] or could have a potential association with acute kidney injury. The caliper width was 0.2 standard deviations of the logit-transformed propensity score. After 1:2 matching, 184 patients with ABO-i LT and 111 patients with ABO-c LT were generated. In our data, ABO-i LT was performed from 2012 to 2018, which was relatively recent compared to ABO-c LT (between 2010 and 2018). We considered the year of surgery as a covariate of our propensity matching to address for this discrepancy.

For descriptive statistics, the incidence of AKI according to pRBCs and FFP were compared between ABO-c and ABO-i LT after matching. To evaluate the association between pRBCs and FFP transfusion amounts and the risk of AKI in the matched cohort, the chi-squared test for trends was performed both in patients with ABO-c and ABO-i after matching. 

In addition, a conditional logistic regression analysis was performed to calculate and compare the odds ratio of transfusion of RBCs and FFP for the risk of AKI in each matched cohort of ABO-c and ABO-i LT (separately). Cubic spline function curves were drawn to evaluate the adjusted relationship between the amount of red blood cell transfusion as a continuous variable and the probability of AKI.

Finally, we compared clinical outcomes other than AKI between ABO-c and ABO-i LT, including the incidence of postoperative hemodialysis and the length of ICU and hospital stays.

## 3. Results 

During the first seven postoperative days, AKI, as determined by the KDIGO criteria, was observed in 342 patients (38.6%) of our retrospective cohort (stage 1, *n* = 251, 28.4%; stages 2 and 3, *n* = 91, 10.3%). Patient demographics and surgery-related variables before and after propensity score matching in both ABO-c and ABO-I LT are shown in Table 1. 

The incidence of AKI in ABO-i LT (*n* = 67/119, 56.3%) was significantly higher than in ABO-c LT (*n* = 275/766, 35.9%, *p* < 0.001) (Supplementary Materials, Figure S1). The incidence of stage 2 or 3 AKI was also significantly higher in ABO-i LT than in ABO-c LT (*n* = 21/119, 17.6% vs *n* = 70/766, 9.1%, *p* = 0.004). 

The results of the multivariable logistic regression analysis for post-transplant AKI are shown in Table 2. ABO-i LT was identified as an independent predictor of AKI (odds ratio (OR) = 2.54, 95% confidence interval (CI) 1.20–5.38, *p* = 0.015). The performance of our multivariable prediction model was fairly good (area under the receiver-operating characteristic curve = 0.73, 95% CI, 0.70–0.77, Negelkerke’s *R*^2^ = 0.219) with good calibration (Hosmer–Lemeshow goodness-of-fit, chi-squared = 8.98, *p* = 0.344). 

The propensity-score-matched sample set comprised 184 cases of ABO-c LT and 111 cases of ABO-i LT. There was no unbalanced contributor with a standardized difference of ≥0.20 (Supplementary Materials, Figure S2). In the propensity-score-matched cohort, the incidence of AKI was still higher in ABO-i LT (*n* = 73/111, 65.8%) than in ABO-c LT (*n* = 73/184, 39.7%, *p* < 0.001). The incidence of stage 2 or 3 AKI was also significantly higher in ABO-i LT (*n* = 35/111, 31.5% vs *n* = 25/184, 13.6%, *p* < 0.001).

Supplementary Tables S1 and S2 show the distribution of the incidence of AKI according to RBC and FFP transfusion in the matched cohort. The incidence of AKI rose in direct proportion to the amount of FFP transfusion, and this increase was more pronounced in ABO-i LT: in ABO-i LT, the incidence increased from 33.3% among those not transfused to 84.0% among those transfused with ≥10 units (chi-squared test for trends, *p* < 0.001), while in ABO-c LT, it increased from 15.2% among those not transfused to 60.0% among those transfused with ≥10 units (chi-squared test for trends, *p* < 0.001). These trends were significantly different between groups (*p* < 0.001).

The expected odds ratio and their 95% CIs of AKI in propensity-score-matched ABO-c and ABO-i LT patients according to the number of pRBC or FFP transfusions are shown in Table 3 and Table 4, respectively. ORs were calculated using conditional logistic regression analyses in a propensity-score-matched cohort of both ABO-c and ABO-i LT patients, separately. The OR of AKI increased as the number of transfusions increased, and this trend was more pronounced in ABO-i LT compared to ABO-c LT. 

**Figure 1:**
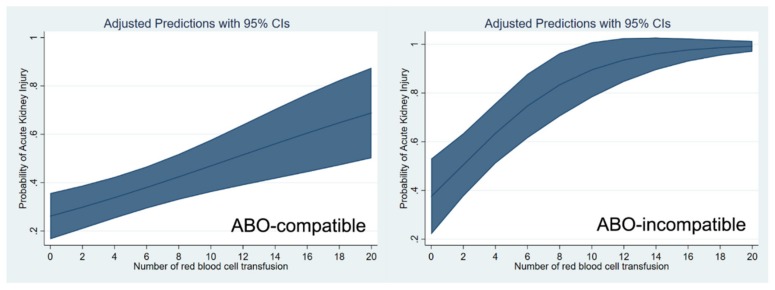
Cubic spline function curve relating the number of packed red blood cell transfusions to the risk of postoperative acute kidney injury in ABO-compatible (left) and ABO-incompatible (right) liver transplantations.

**Figure 2:**
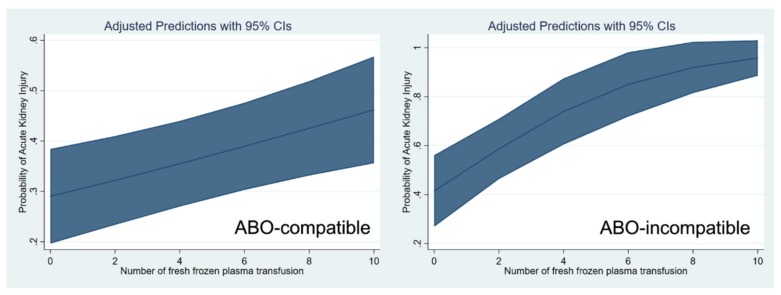
Cubic spline function curve relating the number of fresh frozen plasma transfusions to the risk of postoperative acute kidney injury in ABO-compatible (left) and ABO-incompatible (right) liver transplantations.

These relationships are shown through a cubic spline function curve analysis, which relates the number of pRBC and FFP transfusions to the risk of post-transplant AKI in both ABO-c (Figure 1) and ABO-i LT (Figure 2). The cubic splines were positively sloped, and the slope was steeper in ABO-i LT than in ABO-c LT. The association of transfusion with the risk of AKI was stronger in ABO-i LT compared to ABO-c LT. 

## 4. Discussion

The main finding of our study is that the risk of AKI rose proportionally according to the amount of pRBC and FFP transfusions in both ABO-c and ABO-i LT, but this association of pRBC and FFP transfusions with post-transplant AKI was stronger in ABO-i LT than in ABO-c LT. Given that the AKI rates increased in patients with preoperative anemia and that the influence of transfusion on AKI was more pronounced in ABO-i LT patients, any efforts to reduce perioperative transfusions may protect patients with ABO-i LT against AKI. However, as our observations were from a retrospective analysis, prospective trials are required to confirm our hypothesis.

Patient mortality and graft survival after ABO-i LT have been reported to be comparable to ABO-c LT [16,18,19,20], although there is still concern regarding the high incidence of antibody-mediated rejection, including biliary stricture related to a high isoagglutinin titer [19]. This high isoagglutinin titer may provoke another major organ injury, including AKI, through an immunologic response [30]. As AKI affects graft outcomes and mortality after liver transplantation [3,4,7,12], it is important to elucidate modifiable risk factors of AKI in ABO-i LT. 

The reason why AKI occurs more frequently in ABO-i LT is not certain, although the high isoagglutinin titer may play a role. A high isoagglutinin titer may lead to major organ damage through possible immunologic injury [30]. In addition, the prevalent baseline metabolic alkalosis, low platelet count, and high incidence of mild arterial hypoxemia prior to transplantation surgery may contribute to the high incidence of intraoperative transfusion and low oxygen delivery to the kidney [21]. Furthermore, patients with ABO-i LT already receive a substantial amount of FFP transfusion for plasma exchange prior to surgery. FFP has been reported to be a risk factor for AKI after surgery, including liver transplantation [2,31,32], and the risk of AKI may increase when patients with ABO-i LT receive intraoperative FFP transfusions. Furthermore, an inflammatory, allergic, and immunologic reaction triggered by FFP transfusion may be exacerbated when patients receive additional intraoperative FFP [33,34]. 

Red cell transfusion is also a well-known risk factor of AKI after liver transplantation [2,35]. The negative effect of pRBC transfusion is multifaceted [36,37] and includes a systemic inflammatory response, which contributes to the development of AKI after surgery. A large amount of transfusion reflects excessive surgical bleeding, which leads to renal hypoperfusion and ischemia, resulting in poor oxygen delivery. There was a dose–response relationship between the amount of pRBC and FFP transfusion and the risk of AKI in our study. However, it is not clear why the patients undergoing ABO-i LT were more susceptible to the deleterious effects of pRBC transfusion. Possible mechanisms include that the adverse effect of pRBC transfusion may be aggravated by a high isoagglutinin titer [30]. Although we accounted for confounding factors through propensity-score-matching and multivariable analysis, we could not adjust the isoagglutinin titer due to a lack of data. In addition, red cell transfusion is closely related to FFP transfusion. Cumulative FFP transfusion after preoperative plasma exchange could be the leading cause, and pRBC transfusion could be a simple association.

To reduce the transfusion amount in ABO-i LT, we could adopt a restrictive transfusion policy by decreasing the transfusion threshold. Although there is no established consensus regarding intraoperative transfusion, the pRBC transfusion threshold in our institution during liver transplantation was around 20% in hematocrit [38]. This threshold could be lowered further depending on the baseline status of recipients or the mixed venous oxygen concentrations. Red cell transfusion during surgery could possibly be reduced by optimizing baseline hematocrit before surgery through the administration of erythropoietin. A previous randomized trial reported that a preoperative single intravenous dose of erythropoietin could reduce the transfusion amount during cardiac surgery, thereby reducing the incidence of postoperative AKI [39]. In addition, to reduce transfusion requirements, the intraoperative use of vasopressin was reported to reduce surgical bleeding through selective splanchnic vasoconstriction and by reducing portal blood flow [40].

The incidence of AKI in our study was within the range reported in previous literature [1,2,3,5,6,7,8,9,10]. The incidence of AKI in ABO-i LT was also similar to a previous study [9]. The risk factors of AKI other than ABO-i LT were mostly consistent with those of the previous literature. The preoperative hemoglobin level was independently associated with post-transplant AKI [8,22,35]. The MELD score has been consistently reported to be a predictor of AKI [2,3,9,24]. Ischemic time was not identified as an independent predictor of AKI in our analysis, which could have been due to the relatively short ischemic time of LDLT.

Our study had several important limitations. First, our study had the common drawbacks of a retrospective single-center study. Although we performed a propensity-score-matched analysis with perioperative variables, unknown or unmeasured biases such as the isoagglutinin titer could not be addressed. The external validity was limited. Our perioperative patient care and desensitization protocol may differ from other institutions. Second, the mechanism underlying the association could not be demonstrated. It is not clear whether the increased risk of AKI by transfusion in ABO-i LT was truly the effect of transfusion or whether transfusion was simply a marker for intraoperative procedural complexity. The strong association between transfusion and AKI could also be due to the preoperative conditioning regimen for ABO-i LT. A restrictive or liberal transfusion strategy may also influence our results. However, a causal relationship could not be proven due to the retrospective study design. A prospective clinical trial is required for further evaluation. Well-designed randomized trials may also test the hypothesis that reducing the intraoperative transfusion amount may attenuate the risk of AKI, especially in ABO-i LT. Third, there was a relatively small sample size in the ABO-i LT group. This inevitably resulted in a small number of patients in the matched cohort, limiting the study power of the statistical analysis in the matched cohort. However, although we used many contributors to the propensity score, most of the cases in the ABO-i LT group remained after matching because there was a relatively large number of candidate cases for matching in the ABO-c LT group.

## 5. Conclusions

Our study demonstrated that the incidence of AKI after ABO-i LT was significantly higher than after ABO-c LT, and the risk was higher in patients with preoperative low hematocrit. The risk of AKI was in direct proportion to the number of transfused pRBC or FFP units, and this association was stronger in ABO-i LT than in ABO-c LT. Given that preoperative hematocrit and transfusions are modifiable risk factors, interventions to optimize the hematocrit level before surgery and to reduce perioperative transfusion may mitigate the risk of AKI, especially in patients with ABO-i LT. However, it is not clear whether the stronger association between transfusion and AKI in ABO-i LT was truly the effect of transfusion. The causal relationship and these hypotheses should be evaluated in future prospective randomized trials.

## Figures and Tables

**Table 1:** Patient characteristics and perioperative parameters.

	Before Matching			Standardized Difference	After Matching			Standardized Difference
Characteristic	ABO-i LT	ABO-c LT	*p*-Value		ABO-i LT	ABO-c LT	*p*-Value	
Sample size	119	766			111	184		
Demographic data								
Age, years	55 (49–60)	53 (48–60)	0.054	2.41	54 (49–61)	55 (50–60)	0.691	0.73
Female, *n*	47 (39.5)	176 (23.0)	<0.001	16.52	41 (36.9)	65 (35.3)	0.780	1.61
Body mass index, kg/m^2^	23.1 (21.6–25.4)	23.1 (21.1–25.3)	0.792	0.19	23.2 (21.5–25.3)	23.2 (21.6–25.4)	0.951	0.04
Etiology of liver disease								
Alcoholic liver cirrhosis, *n*	19 (16.0)	70 (9.1)	0.021	6.82	17 (15.3)	31(16.8)	0.374	1.50
HBV hepatitis, *n*	70 (58.8)	328 (42.8)	0.001	16.00	60 (54.1)	95 (51.6)	0.686	2.50
HCV hepatitis, *n*	8 (6.7)	58 (7.6)	0.853	0.85	8 (7.2)	12 (6.5)	0.644	0.70
Hepatocellular carcinoma, *n*	71 (59.7)	420 (54.8)	0.324	4.83	63 (56.8)	106 (57.6)	0.904	0.80
Cholestatic disease, *n*	13 (10.9)	16 (2.1)	<0.001	8.84	12 (10.8)	10 (6.4)	0.121	6.50
Nonalcoholic steatohepatitis, *n*	3 (2.5)	41 (5.4)	0.256	2.83	3 (2.7)	8 (4.3)	0.264	1.60
Baseline medical status								
Hypertension, *n*	17 (14.3)	77 (10.1)	0.163	4.23	16 (14.4)	26 (14.1)	0.284	0.30
Diabetes mellitus, *n*	19 (16.0)	107 (14.0)	0.562	2.00	19 (17.1)	32 (17.4)	0.789	0.30
Preoperative hemoglobin, g/dL	10.4 (8.9–12.1)	10.8 (9.2–12.6)	0.202	0.73	10.4 (8.7–12.4)	11.0 (9.1–12.5)	0.335	0.26
Preoperative serum sodium concentration, mEq/L	139 (134–141)	137 (132–140)	0.050	3.82	141 (137–144)	140 (137–142)	0.180	0.32
MELD score	14.3 (11.1–20.7)	15.9 (12.0–22.7)	0.042	3.09	15.0 (9.9–20.4)	14.2 (9.9–19.7)	0.601	0.60
Child class, *n* (A/B/C)	53 (43.3)/42 (35.3)/ 24 (20.2)	298 (38.9)/280 (36.6)/188 (24.5)	0.194	-	45 (40.5)/ 42 (37.8)/24 (21.6)	75 (40.8)/ 71 (38.6)/38 (20.7)	0.117	-
Preoperative LVEF, %	66 (62–68)	65 (62–68)	0.978	0.46	66 (61–69)	65 (61–69)	0.540	0.35
Preoperative beta-blocker, *n*	7 (5.9)	38 (5.0)	0.670	0.92	7 (6.3)	11 (6.0)	0.273	0.30
Preoperative diuretics, *n*	6 (17.1)	29 (3.8)	0.003	13.4	5 (12.2)	18 (9.8)	0.131	2.40
Previous abdominal surgery, *n*	2 (1.7)	24 (3.1)	0.383	1.45	2 (1.8)	5 (2.7)	0.491	0.90
Donor/graft factors								
Age, years	32 (24–42)	30 (23–39)	0.130	2.88	30 (23–40)	30 (23–38)	0.242	1.16
Estimated GRWR	1.24 (1.05–1.46)	1.20 (1.04–1.41)	0.393	0.02	1.24 (1.02–1.45)	1.21 (1.06–1.47)	0.455	0.00
Operation and anesthesia details								
Operation time, h	6.4 (5.6–7.8)	6.9 (5.8–8.0)	0.074	1.55	6.5 (5.5–7.5)	6.6 (5.4–7.6)	0.093	0.38
Cold ischemic time, min	86 (70–106)	88 (68–240)	0.061	13.69	86 (70–106)	88 (69–109)	0.233	3.40
Warm ischemic time, min	28 (26–33)	30 (28–35)	0.101	4.30	28 (25–33)	30 (26–34)	0.109	1.47
Intraoperative dose of epinephrine bolus, mcg	0 (0–20)	0 (0–30)	0.063	3.12	0 (0–20)	0 (0–10)	0.787	0.65
Reperfusion syndrome, *n*	9 (7.6)	79 (10.3)	0.351	2.75	9 (8.1)	12 (6.5)	0.644	1.60
Intraoperative mean blood glucose, mg/dL	155 (152–161)	163 (145–180)	<0.001	9.37	157 (154–164)	160 (148–170)	0.092	3.61
Crystalloid intake, 100 mL	33 (22–49)	36 (25–53)	0.023	0.57	34 (23–48)	34 (23–45)	0.646	0.40
Colloid intake, 100 ml	0 (0–0)	0 (0–5)	0.001	2.55	0 (0–3)	0 (0–4)	0.102	0.68
Bleeding and transfusion amount								
pRBC transfusion, units	5 (2–10)	6 (2–12)	0.048	1.25	5 (0–9)	5 (0–10)	0.942	0.02
FFP transfusion, units	5 (1–9)	6 (1–12)	0.060	3.47	3 (0–7)	3 (0–8)	0.101	0.98
Blood loss per body weight, mL/kg	42 (22–86)	47 (24–100)	0.056	22.11	36 (19–73)	36 (18–73)	0.753	9.29

The values are expressed as the median (interquartile range) or number (%). LT = liver transplantation; ABO-c = ABO-compatible; ABO-I = ABO-incompatible; MELD score = “Model for End-Stage Liver Disease” score; LVEF = left ventricular ejection fraction; GRWR = graft recipient body weight ratio; pRBCs = packed red blood cells; FFP = fresh frozen plasma.

**Table 2:** Multivariable logistic regression analysis for acute kidney injury (AKI) defined by “Kidney Disease Improving Global Outcomes” (KDIGO) criteria after liver transplantation in all patients (*n* = 885).

Variable	Odds Ratio	95% CI	*p*-Value
Body mass index, recipient	1.07	1.01–1.12	0.012
Preoperative beta-blocker administration	2.00	0.86–4.14	0.052
Preoperative diuretics administration	1.89	0.86–4.14	0.113
ABO-incompatible liver transplantation	2.54	1.20–5.38	0.015
MELD score	1.02	1.00–1.04	0.049
Preoperative hemoglobin, g/dL	0.89	0.82–0.97	0.007
Postreperfusion syndrome	1.09	0.99–1.80	0.051
pRBC transfusion			
As continuous variable, per unit	1.09	1.06–1.11	<0.001
No red cell transfusion	Reference		
1–2 units	1.07	0.97–2.04	0.050
3–4 units	1.92	1.11–2.41	0.015
5–9 units	3.20	1.95–5.44	<0.001
≥10 units	4.88	3.00–6.84	<0.001

MELD score = “Model for End-Stage Liver Disease Score”; LVEF = left ventricular ejection fraction; GRWR = graft recipient body weight ratio; pRBCs = packed red blood cells; CI: confidence interval. A backward-Wald variable selection process was used with a significance criterion of 0.20.

**Table 3:** Odds ratios of red cell transfusion for acute kidney injury defined by KDIGO criteria after liver transplantation by conditional logistic regression analysis in the matched cohort of ABO-compatible and ABO-incompatible LT groups.

Variable	Odds Ratio	95% CI	*p*-Value
ABO-compatible LT in the matched cohort			
Red cell transfusion as continuous variable	1.11	1.04–1.17	0.001
Red cell transfusion as categorized variable			
No transfusion	Reference		
1–2 units	1.73	0.96–2.35	0.068
3–4 units	2.01	1.04–3.27	0.040
5–9 units	3.29	1.73–5.02	0.015
≥10 units	4.45	2.37–6.61	<0.001
ABO-incompatible LT in the matched cohorts			
Red cell transfusion as continuous variable	1.32	1.13–1.59	<0.001
Red cell transfusion as categorized variable			
No transfusion	Reference		
1–2 units	1.94	1.04–2.64	0.010
3–4 units	3.05	1.43–5.29	<0.001
5–9 units	4.67	2.44–6.84	<0.001
≥10 units	5.99	3.84–8.01	<0.001

LT = liver transplantation; CI = confidence interval.

**Table 4:** Odds ratios of fresh frozen plasma transfusions for acute kidney injury defined by KDIGO criteria after liver transplantation by conditional logistic regression analysis in the matched cohort of ABO-compatible and ABO-incompatible LT groups.

Variable	Odds Ratio	95% CI	*p*-Value
ABO-compatible LT in the matched cohort			
Fresh frozen plasma as continuous variable	1.07	1.03–1.13	0.002
Fresh frozen plasma as categorized variable			
No transfusion	Reference		
1–5 units	2.41	1.27–4.15	0.012
5–9 units	3.46	1.34–6.19	0.004
≥10 units	4.68	1.81–8.01	<0.001
ABO-incompatible LT in the matched cohorts			
Fresh frozen plasma as continuous variable	1.44	1.16–1.75	<0.001
Fresh frozen plasma as categorized variable			
No red cell transfusion	Reference		
1–5 units	3.59	2.27–5.71	<0.001
6–9 units	4.86	2.92–7.22	<0.001
≥10 units	5.98	3.02–9.65	<0.001

LT = liver transplantation; CI = confidence interval.

## Supplementary Materials

The following are available online at https://www.mdpi.com/2077-0383/8/11/1785/s1. Section 1: Anesthesia, surgical technique, immunosuppression, and preoperative desensitization preparation; Table S1: The incidence of acute kidney injury (AKI) according to the number of red cell transfusions in ABO-compatible and ABO-incompatible liver transplantations in the matched cohort; Table S2: The incidence of acute kidney injury (AKI) according to the number of fresh frozen plasma transfusions in ABO-compatible and ABO-incompatible liver transplantations in the matched cohort; Figure S1: The incidences of acute kidney injury at stages according to the KDIGO criteria between ABO-compatible (ABO-c) and ABO-incompatible (ABO-i) liver transplantation before (upper) and after (lower) propensity score matching; Figure S2: Histograms (left) and covariate balance plot (right) of the distribution of standardized differences in the covariates between the patients with ABO-compatible and ABO-incompatible liver transplantations.
