# Supplementary material for: Influence of Transfusion on the Risk of Acute Kidney Injury: ABO-Compatible versus ABO-Incompatible Liver Transplantation
Source: J Clin Med. 2019 Oct 25;8(11):1785. doi: 10.3390/jcm8111785 (PMC6912207; doi:10.3390/jcm8111785)
Supplement: Supplementary file 1 [file jcm-08-01785-s001.pdf]

	Title	Page
**Section 1**	Anesthesia, surgical technique, immunosuppression, and preoperative desensitization preparation.	2
**Table S1**	The incidence of acute kidney injury (AKI) according to the number of red cell transfusions in ABO-compatible and ABO-incompatible liver transplantations in the matched cohort.	4
**Table S2**	The incidence of acute kidney injury (AKI) according to the number of fresh frozen plasma transfusions in ABO-compatible and ABO-incompatible liver transplantations in the matched cohort.	5
**Figure S1**	The incidences of acute kidney injury at stages according to the KDIGO criteria between ABO-compatible (ABO-c) and ABO-incompatible (ABO-i) liver transplantations before (upper) and after (lower) propensity score matching.	6
**Figure S2**	Histograms (left) and covariate balance plot (right) of the distribution of standardized differences in the covariates between the patients with ABO-compatible and ABO-incompatible liver transplantations.	7

1. Anesthesia, Surgical Technique, Immunosuppression, and Preoperative Desensitization Preparation

Anesthesia for liver transplantation surgery was maintained with propofol with remifentanil. Volume-controlled ventilation was maintained at a tidal volume of 6–8 mL/kg. Arterial-line catheters were inserted into the radial and femoral arteries. Continuous cardiac index and right ventricle-associated variables were monitored. Ephedrine and the continuous infusion of dopamine and/or norepinephrine and/or epinephrine were used to treat hypotension according to the monitored cardiac index, mixed venous oxygen saturation, and systemic vascular resistance. The intraoperative red blood cell transfusion threshold was consistent at 20% in hematocrit during the study period. A histidine–tryptophan–ketoglutarate solution was used for donor grafts. The piggyback technique was used to anastomose the graft and donor vessels. End-to-end anastomosis of the hepatic artery and duct-to-duct anastomosis of the bile duct were performed in succession.

All patients undergoing ABO-i LDLT received a single intravenous dose of rituximab (300–375 mg/m^2 body surface area) 2–3 weeks prior to surgery. All ABO-i LDLT recipients' anti-ABO isoagglutinin titers were assessed at admission, at each round of plasma exchange, on the day before the surgery, and in the postoperative period. The timing and frequency of assessment were readjusted depending on the isoagglutinin level. For plasma exchange, blood type AB fresh-frozen plasma was used. A plasma exchange was performed to achieve an isoagglutinin titer of 1:8 or less before the surgery and was continued until this desired titer was achieved. During the anhepatic period, intravenous methylprednisolone was administered at a dose of 10 mg/kg just prior to reperfusion, before being switched to oral methylprednisolone at a dose of 0.5 mg/kg/day (tapered over 3 months after ABO-i LT). Immunosuppression after ABO-i LT was comprised of corticosteroid, tacrolimus, and mycophenolate mofetil (0.5–1.5 g/day) [1, 2]. The immunosuppressive regimen for patients undergoing ABO-c LT consisted of basiliximab induction, corticosteroid, and tacrolimus. In patients with decreased renal function (ABO-c LDLT recipients), mycophenolate mofetil was used in combination with a reduced dosage of tacrolimus. Corticosteroid was generally tapered off over 3 months after LT [1].

References:

1 Song GW, Lee SG, Hwang S, et al. Biliary stricture is the only concern in ABO-incompatible adult living donor liver transplantation in the rituximab era. *J Hepatol* 2014; 61: 575-82.

2 Song GW, Lee SG, Hwang S, et al. ABO-Incompatible Adult Living Donor Liver Transplantation Under the Desensitization Protocol With Rituximab. *Am J Transplant* 2016; 16: 157-70.

Table S1. The incidence of acute kidney injury (AKI) according to the number of red cell transfusions in ABO-compatible and ABO-incompatible liver transplantations in the matched cohort.

Number of pRBC Transfusions	0 units	1–2 unit(s)	3–4 units	5–9 units	>10 units	*p*-Value [1]	*p*-Value [2]
AKI in ABO-c LT (*n* = 184)	7/46 (15.2)	6/18 (33.3)	12/31 (38.7)	18/39 (46.2)	30/50 (60.0)	<0.001	<0.001
AKI in ABO-i LT (*n* = 111)	10/30 (33.3)	6/11 (54.5)	10/13 (76.9)	26/32 (81.3)	21/25 (84.0)	<0.001	

Data are presented as numbers (%). ABO-i = ABO-incompatible; ABO-c = ABO-compatible; *p*-value [1] tests the trend within each group; *p*-value [2] compares trends between groups.

Table S2. The incidence of acute kidney injury (AKI) according to the number of fresh frozen plasma transfusions in ABO-compatible and ABO-incompatible liver transplantations in the matched cohort.

Number of FFP Transfusions	0 units	1–5 unit(s)	6–9 units	≥10 units	*p*-Value [1]	*p*-Value [2]
AKI in ABO-c LT (*n* = 184)	13/66 (19.7)	17/38 (44.7)	19/37 (51.4)	24/43 (55.8)	0.001	<0.001
AKI in ABO-i LT (*n* = 111)	18/44 (40.9)	23/30 (76.7)	15/18 (83.3)	17/19 (89.5)	<0.001	

Data are presented as numbers (%). ABO-i = ABO-incompatible; ABO-c = ABO-compatible; *p*-value [1] tests the trend within each group; *p*-value [2] compares trends between groups.

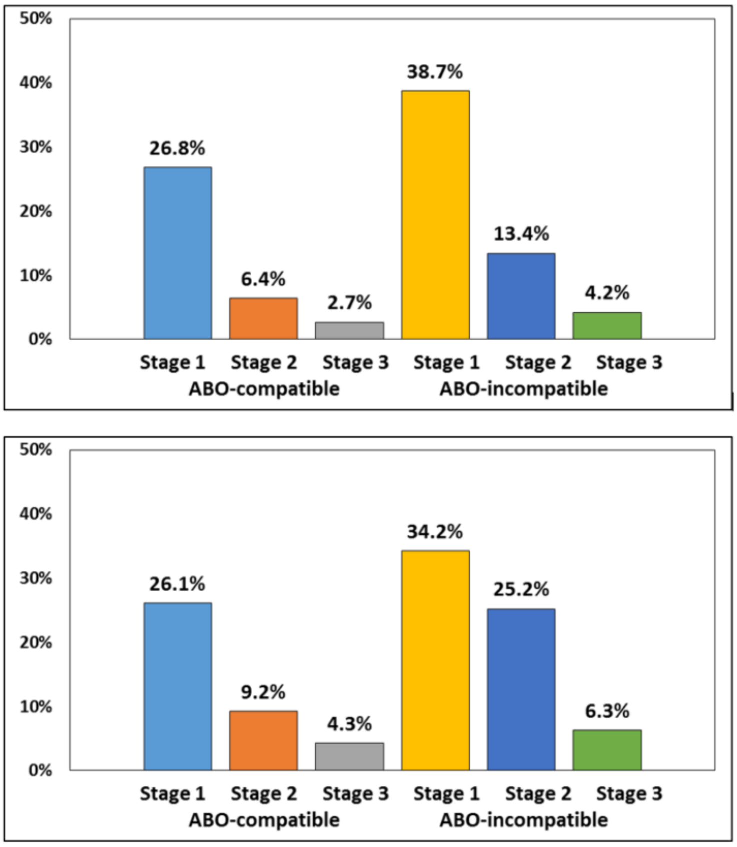

Figure S1. The incidences of acute kidney injury (AKI) at stages according to the KDIGO criteria between ABO-compatible and ABO-incompatible liver transplantations before (upper) and after (lower) propensity score matching. The incidence of AKI was significantly higher in ABO-incompatible liver transplantation than in ABO-compatible liver transplantation before ($p < 0.001$) and after ($p < 0.001$) propensity score matching. The incidence of stage 2 or 3 AKI was also significantly higher in ABO-incompatible liver transplantation than in ABO-compatible liver transplantation before ($p = 0.010$) and after ($p < 0.001$) propensity score matching.

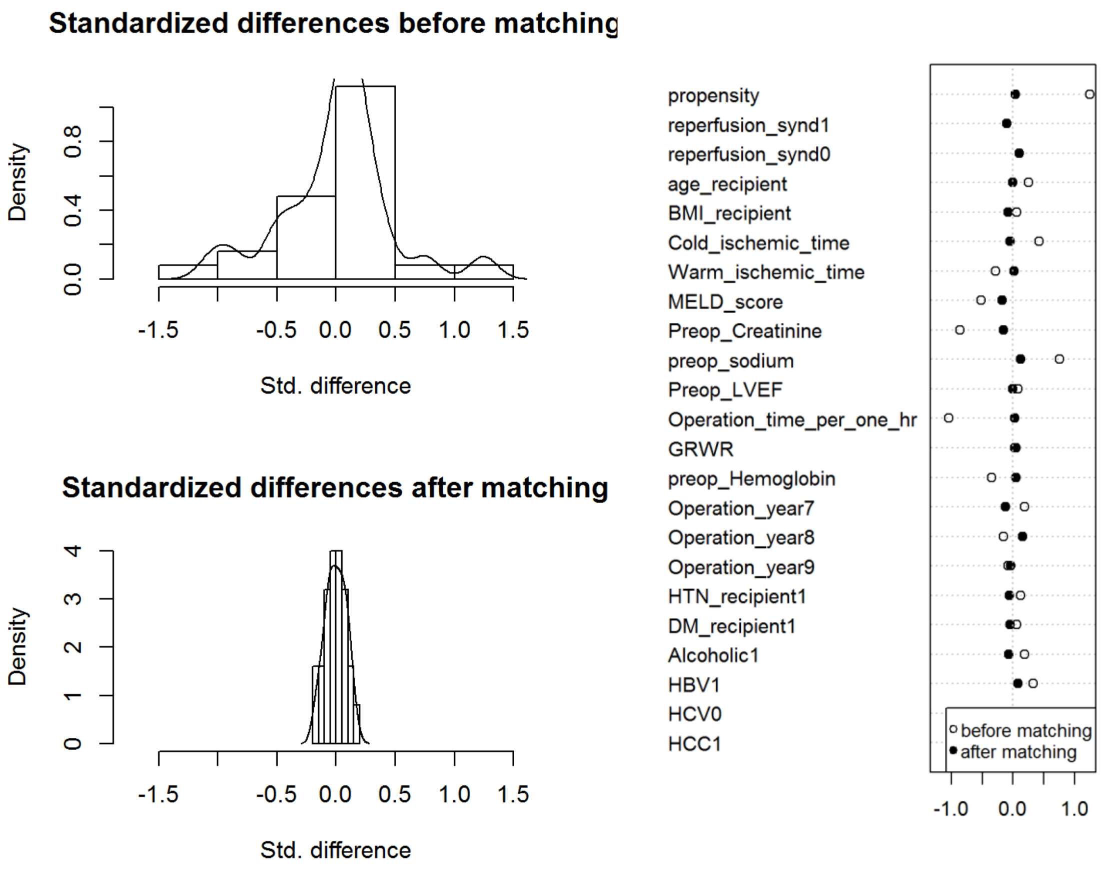

Figure S2. Histograms (left) and covariate balance plot (right) of the distribution of standardized differences in the covariates between the patients with ABO-compatible and ABO-incompatible liver transplantations. BMI = body mass index; MELD score = "Model for End-Stage Liver Disease" score; Preop_LVEF = preoperative left ventricle ejection fraction; GRWR = graft recipient body weight ratio; HTN = hypertension; DM = diabetes mellitus; Alcoholic = alcoholic liver cirrhosis; HBV = hepatitis B; HCV = hepatitis C; HCC = hepatocellular carcinoma.
